# Syncope due to recurrent ventricular tachycardias after transcatheter aortic valve implantation with unexpected diagnosis in cardiac computed tomography: a case report

**DOI:** 10.1093/ehjcr/ytae300

**Published:** 2024-06-12

**Authors:** Philipp Breitbart, Hannah Billig, Florian André, Norbert Frey, Grigorios Korosoglou

**Affiliations:** Department of Cardiology and Angiology, Medical Center-University of Freiburg, Faculty of Medicine, University of Freiburg, Südring 15, 79189 Bad Krozingen, Germany; Medical Department II, University Hospital Bonn, Bonn, Germany; Department of Cardiology, Angiology and Pneumology, Heidelberg University Hospital, Heidelberg, Germany; DZHK (German Centre for Cardiovascular Research), Partner site Heidelberg, Germany; Department of Cardiology, Angiology and Pneumology, Heidelberg University Hospital, Heidelberg, Germany; DZHK (German Centre for Cardiovascular Research), Partner site Heidelberg, Germany; GRN Hospital Weinheim, Department of Cardiology, Vascular Medicine & Pneumology, Weinheim, Germany; Weinheim Cardiac Imaging Center, Hector Foundation, Weinheim, Germany

**Keywords:** Echocardiography, Cardiac computed tomography angiography (CCTA), Transfemoral aortic valve implantation (TAVI), Case report

## Abstract

**Background:**

Delayed coronary obstruction (DCO) is a rare but potentially life-threatening complication after transcatheter aortic valve implantation (TAVI) mostly affecting the left main coronary artery (LMCA) and often caused by prosthesis endothelialization or thrombus formations. Herein, we report an unusual case of a delayed LMCA-obstruction caused by a calcium nodule, which was diagnosed 4 months after TAVI due to recurrent ventricular tachycardia (VT) episodes.

**Case summary:**

A 73-year-old patient was readmitted to an external hospital with syncope three months after TAVI. Fast VT could be induced in electrophysiological examination, why the patient received a two-chamber implantable cardioverter defibrillator (ICD). However, after 1 month the patient was readmitted to our department with another syncope. Implantable cardioverter defibrillator records revealed multiple fast VT episodes (200–220 b.p.m.). In addition, the patient reported new-onset exertional dyspnoea (New York Class Association Stage III) and elevated high-sensitive cardiac troponin of 115 ng/L. Due to the symptoms and laboratory markers indicating potential myocardial ischaemia, a cardiac computed tomography angiography (CCTA) was performed. Cardiac computed tomography angiography revealed obstruction of the LMCA likely caused by calcium shift during TAVI. After CCTA-guided percutaneous coronary intervention, patient’s course remained uneventful.

**Discussion:**

The present case report highlights the role of CCTA as a powerful non-invasive diagnostic tool in complex settings after TAVI. Delayed coronary obstruction as a procedural complication can occur after TAVI and manifest with various symptoms, including new-onset or recurrent VTs, like in the present case. Cardiac computed tomography angiography provided accurate assessment of the implanted prosthesis and detection of DCO, thus guiding the subsequent PCI.

**Learning points**

- To point out diverse causes of cardiac arrhythmias, causing syncope in patients with cardiac, valvular, and/or coronary artery disease (CAD).
- To underline the value of cardiac computed tomography for establishing the correct diagnosis and guiding appropriate therapeutic decisions after transcatheter aortic valve implantation in patients with concomitant CAD.

## Introduction

Delayed coronary obstruction (DCO) is a rare but potentially life-threatening complication after transcatheter aortic valve implantation (TAVI) mostly affecting the left main coronary artery (LMCA) and often caused by prosthesis endothelialization or thrombus formations, and more commonly occurring after valve-in-valve procedures.^1^ Herein, we report an unusual case of a delayed LMCA-obstruction caused by a calcium nodule, which was diagnosed 4 months after TAVI due to recurrent ventricular tachycardia (VT) episodes.

## Summary figure

**Figure:**
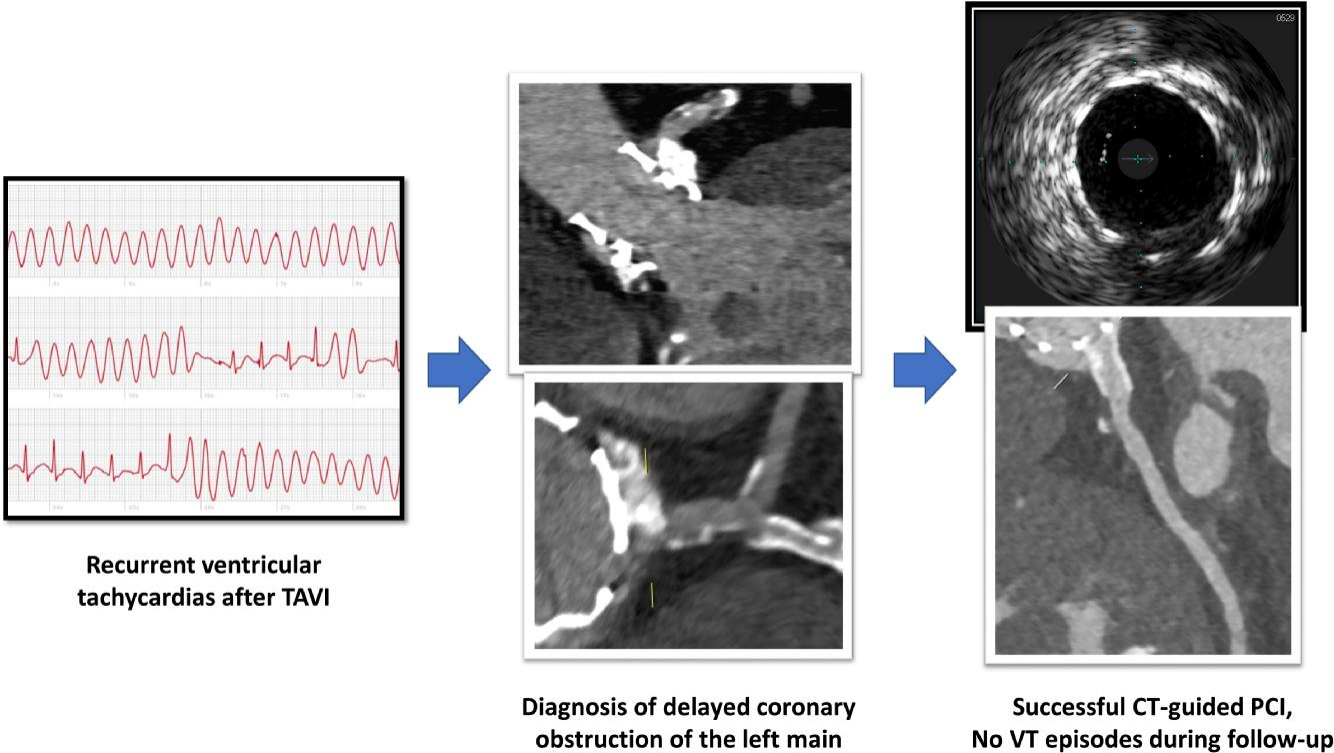


## Case presentation

We describe a case of a 73-year-old female patient, presenting with symptomatic heart failure [New York Class Association (NYHA) IV] and resting dyspnoea at our emergency room.

On physical examination, blood pressure was reduced (100/60 mmHg), heart rate was elevated (110 b.p.m.) (shock index = 1.1), and oxygen saturation was 80%. Heart auscultation revealed a low intensity mid-systolic murmur, electrocardiogram exhibited sinus tachycardia.

The patient had history of hypertension and type 2 diabetes mellitus but no history of cardiac diseases.

Echocardiography revealed global systolic heart failure with moderately reduced left ventricular ejection fraction (LVEF) of 38%. In addition, severe aortic valve stenosis was diagnosed (max. gradient of 90 mmHg, mean gradient of 55 mmHg, and aortic valve area of 0.7cm²).

Laboratory chemical diagnostics including cardiac, kidney, and liver parameter revealed elevated high-sensitive cardiac troponin of 710 ng/L and NT-pro BNP of 9522 pg/mL. Cardiac troponin remained stable with a value 723 ng/L at 2 h.

Due to congestive heart failure with respiratory failure the patient was transferred to the intensive care unit and received non-invasive ventilation.

Due to the diagnosis of non-ST elevation myocardial infarction and severe symptomatic aortic stenosis, cardiac catheterization was performed. Because of severe coronary lesions, she underwent percutaneous coronary intervention (PCI) of the left anterior descending artery and first diagonal branch (RD1) using DK-crush technique. The patient was further referred for transfemoral TAVI. Cardiac computed tomography angiography (CCTA) showed severe valve calcifications without abnormalities of TAVI planning measurements. The procedure was performed using a balloon-expandable valve (Edwards 26 mm). Control echocardiography after TAVI and PCI, revealed almost normalized LVEF of 50%.

Three months after TAVI, the patient was readmitted to an internal department of an external hospital with syncope. An electrophysiological study was performed, where fast VT could be induced. Therefore, the patient received a two-chamber implantable cardioverter defibrillator (ICD) and was discharged.

However, after 1 month, the patient was readmitted to our department with another syncope. Implantable cardioverter defibrillator records revealed multiple fast VT episodes (200–220 b.p.m.). In addition, the patient reported new-onset exertional dyspnoea (NYHA Stage III) and elevated high-sensitive cardiac troponin of 115 ng/L. Echocardiography revealed normal LVEF.

**Figure 1:**
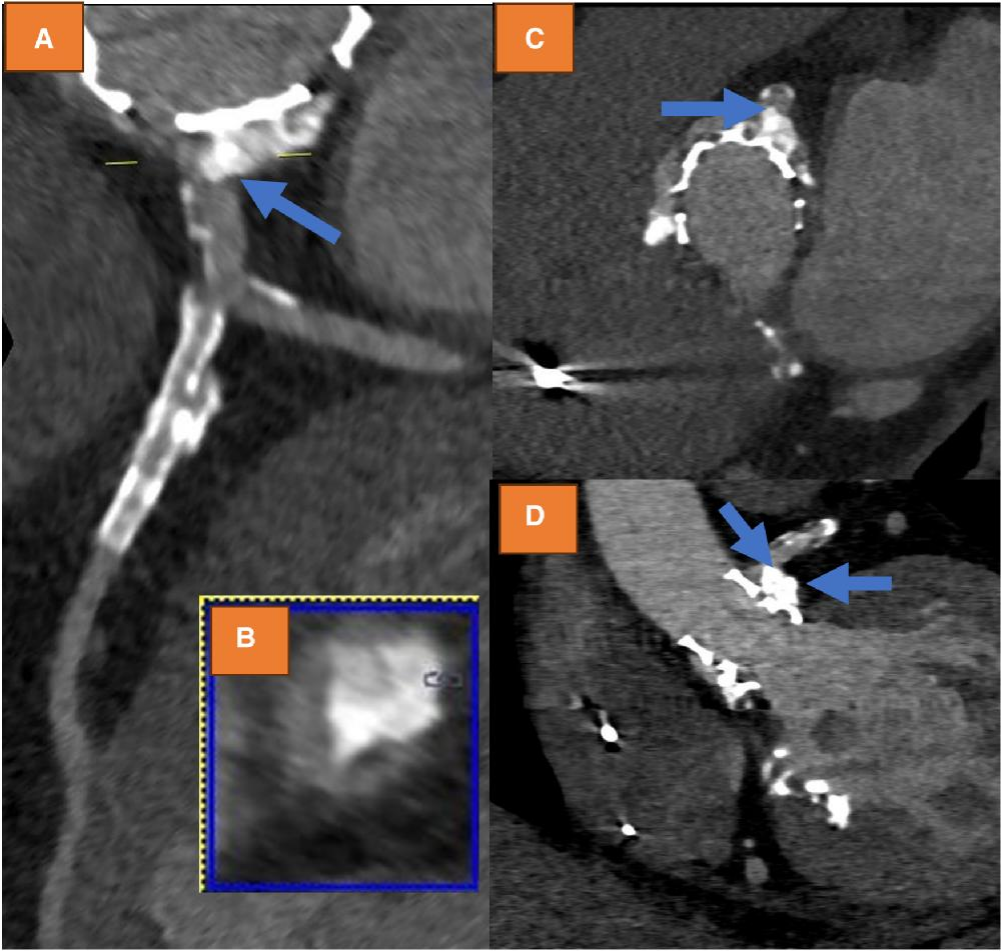
(*A*) Axial, (*B*) circumferential, (*C*) sagittal, and (*D*) coronal cardiac computed tomography angiography images of after the transcatheter aortic valve implantation procedure, demonstrating left main compression by a calcium nodule, causing high-grade left main obstruction.

**Figure 2:**
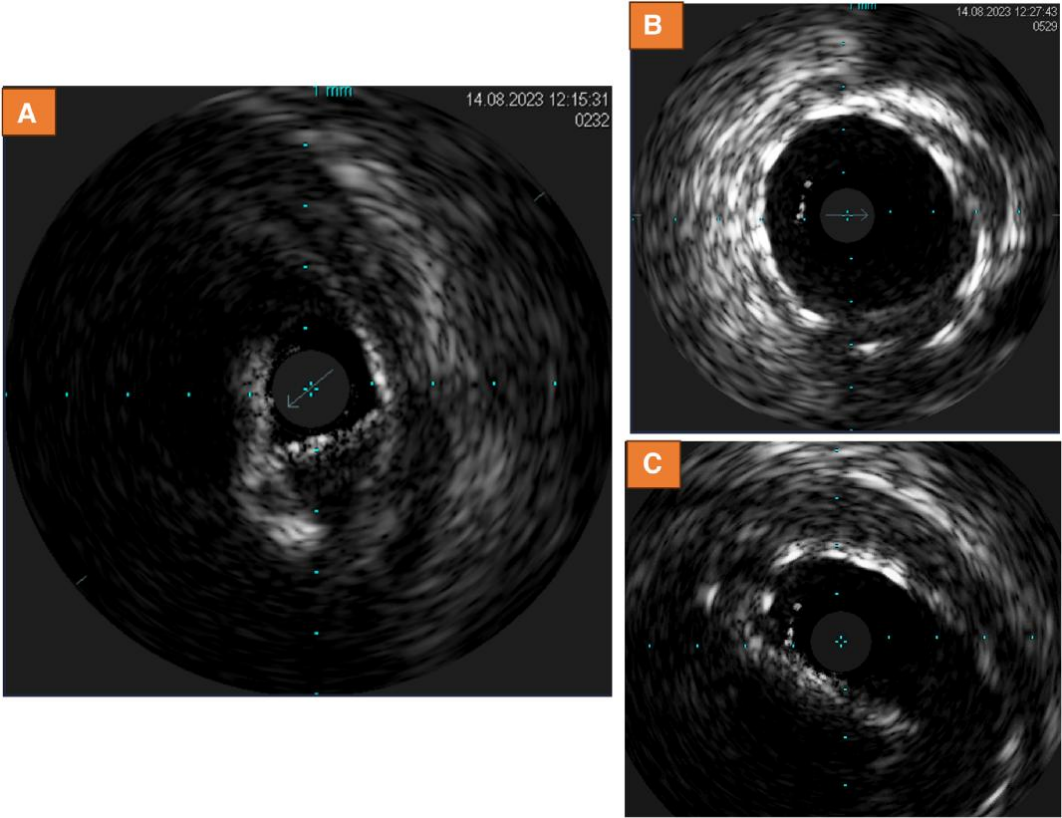
High-grade obstruction of left main coronary artery ostium prior to stent placement (*A*) with a minimal are of 4 mm². After stent implantation, significant lumen gain was observed (*B*) with eccentric stent configuration in the left main coronary artery ostium due to remaining external compression (*C*).

**Figure 3:**
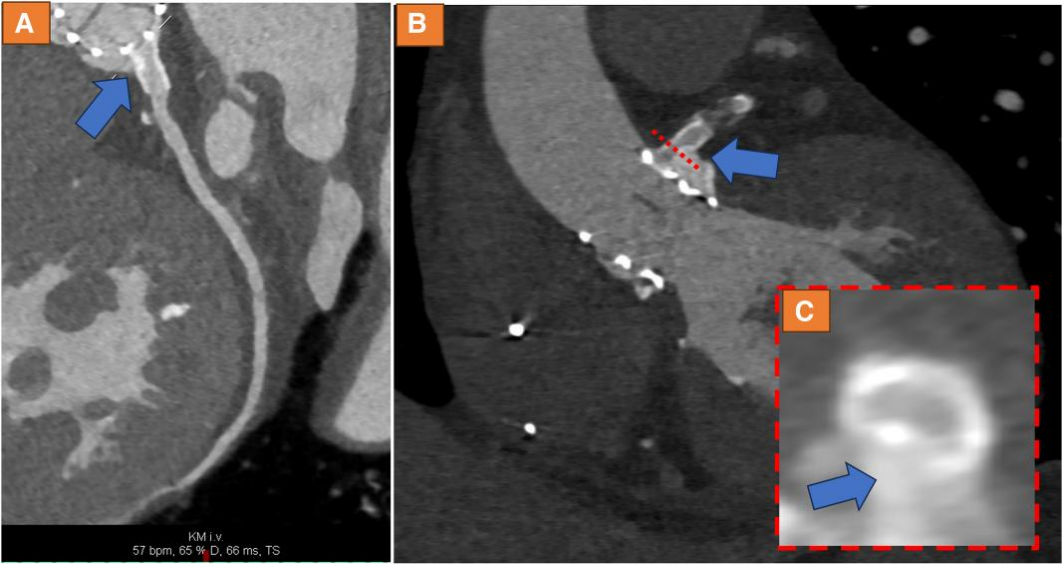
Cardiac computed tomography angiography after the procedure confirmed good lumen gain of the left main coronary artery (*A*), confirming the remaining external compression by valvular calcium (*B–C*).

**Figure 4:**
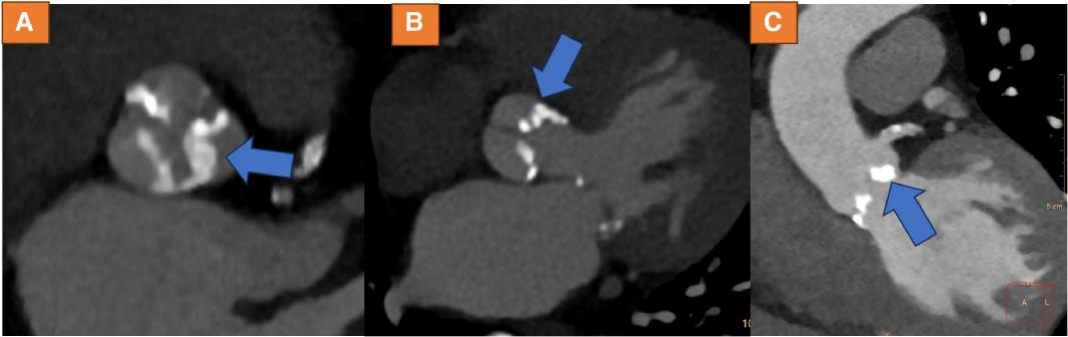
Cardiac computed tomography angiography images before transcatheter aortic valve implantation, demonstrating strong calcification of the left coronary cusp, which possibly shifted towards the left main coronary artery ostium during transcatheter aortic valve implantation (arrows in *A*–*C*).

Due to symptoms and laboratory markers indicating potential myocardial ischaemia, a CCTA was performed. Cardiac computed tomography angiography indeed revealed LMCA compression and obstruction by a calcium nodule, which possibly shifted towards the left main ostium during the TAVI procedure, causing high-grade stenosis (*Figure 1A–C*). Subsequently, cardiac catheterization was performed, confirming the new-onset high-grade ostial LMCA-stenosis (see Supplementary material online, *Video S1A* and *B*). A drug-eluting 5.0*12 mm stent was implanted (see Supplementary material online, *Video S2A*) with good angiographic result (see Supplementary material online, *Video S2B*–*D*). Using intravascular ultrasound, high-grade obstruction was verified prior to stent placement (*Figure 2A*) with a minimal area of 4.0mm², whereas after stenting, significant lumen gain was observed (*Figure 2B*) (minimal area of 10.1 mm²). However, at the LMCA ostium, eccentric stent configuration was noted, which remained after post-dilatation (*Figure 2C*). Cardiac computed tomography angiography after procedure confirmed good lumen gain of LMCA (*Figure 3A*), demonstrating remaining mild external compression by valvular calcium (*Figure 3B* and *C*). Review of CCTA prior to TAVI revealed strong calcification of the left coronary cusp, which possibly shifted towards the left main ostium during TAVI (blue arrows *Figure 4A–C*).

Implantable cardioverter defibrillator records three months after left main stenting revealed no VT episodes and the patient remained uneventful during 1-year clinical follow-up.

## Discussion

Echocardiography after patient examination represents the first line tool for the diagnosis of severe aortic stenosis and routine surveillance examinations after TAVI.^2^ In addition, CCTA allows 3D visualization of the aortic valve annulus and is therefore the imaging method of choice for planning the TAVI procedure and reducing complications.^3,4^ Particular attention is required for the landing zone calcification and coronary ostial height, to avoid coronary occlusion that may be caused by stent frames or shifted calcific nodules, like in our case.^3^ Repeat CCTA post-TAVI enables the assessment of the final prosthesis position, and the detection of specific complications, such as leaflet thromboses or contained ruptures of the aortic annulus.^5–8^ Delayed coronary obstruction with an incidence of 2.2% after TAVI, leading to myocardial ischaemia as one of the most common reasons for recurrent VTs or cardiac arrest, can be caused both as a result of some of these complications directly by the TAVI-prosthesis stent frames or, like in our case, due to a periprocedural calcium shift.^1,9^ Delayed coronary obstruction prognosis is poor with overall in-hospital death rates of 50%.^1^ Notably, the versatility of CCTA with modern generation scanners provides both the evaluation of DCO and underlying causes and concomitant assessment of coronary artery disease (CAD) progression.^10^

In our patient, indeed both exclusion of CAD progression and in-stent-restenosis and DCO-detection after TAVI could be achieved by CCTA. Implantable cardioverter defibrillator implantation was performed due to VT-induction during electrophysiological examination, which again underlines the importance of differential diagnosis in case new-onset VTs, since ICD could have been avoided, if repeated CCTA or coronary angiography had been performed in the first place. In addition, our case highlights the importance of intravascular imaging for ostial coronary lesions and for the evaluation of stent expansion during PCI. Intravascular ultrasound and CCTA after PCI clearly indicated the eccentricity of the implanted stent due to remaining external compression, which was not so clearly seen by angiographic images. Finally, direct cardiac catheterization would also have been an acceptable diagnostic pathway in this case.

The present case report highlights the role of CCTA as a powerful non-invasive diagnostic tool in complex settings after TAVI. Delayed coronary obstruction as a procedural complication can occur after TAVI and manifest with various symptoms, including new-onset or recurrent VTs, like in the present case. Cardiac computed tomography angiography provided accurate assessment of the implanted prosthesis and detection of DCO, thus guiding the subsequent PCI.

## Lead author biography


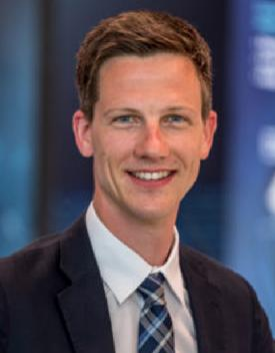


Senior Physician and interventional cardiologist at the University Heart Center Freiburg - Bad Krozingen, Germany. Certified with the highest degree of Cardiac-MRI and Cardiac-CT of the German Cardiac Society. Chair of Young DGK.

## Supplementary Material

ytae300_Supplementary_Data

## Data Availability

All data are incorporated into the article and its Supplementary material online.

## Notes

**Consent:** The patient gave written informed consent for publication of the case. The patient's completed consent form will be retained by the treating facility in accordance with locally approved procedures and stored with the patient's medical records. We hereby confirm compliance with the COPE guidelines.

**Funding:** No funding was received for this manuscript.
